# Miscellaneous

**Published:** 1888-04

**Authors:** 


### MISCELLANEOUS.

CHICAGO'S NEW GARBAGE CREMATORY.—It has been started and it does its work to the satisfaction of the Health Department. Only one of the furnaces was started up for work at 9 o'clock yesterday morning, and the other three were started about the same time for the purpose of drying and getting them in shape for work. The one that was started proved ample to dispose of the garbage on hand until some twenty carts pulled up all at once. Then there was a slight delay before all of them could be emptied.

It is situated on Grant avenue, in the western part of the city, in a stone-quarry, and the top is just on a level with the street. The garbage wagons drive on top of it and the loads are dumped into schutes that land them on what are at present temporary floorings. From there they are shoveled through doors into the furnace. They fall on a grating and over this the flames pass.

The furnaces are at the north and south ends of the building and the chimney is in the middle. The draft thus carries the flames over all the garbage dumped into the ovens and the heat consumes it. The ashes as they are formed drop through the grating into the ash-pans beneath. And so the garbage of the city will be destroyed in the future.

The one oven working did excellently well. Garbage of all kinds was dumped into it, and went into ashes in an incredibly short space of time. The other furnaces will be under way soon. The floors have not yet been constructed for them, and until that is done it will be impossible to put garbage into them. The cost of the furnace was $10,000.

THE ANNUAL MEETING AND BANQUET OF THE ALUMNI ASSOCIATION OF THE INTERNES OF COOK COUNTY HOSPITAL.—The annual meeting of this association occurred March 28, 1888, at the hospital. By invitation, Professor Christian Fenger delivered an address on struma. Dr. Winslow, of Jacksonville, Illinois, was elected president, and Dr. C. E. Caldwell, of Chicago, vice-president, for the ensuing year. Dr. Harris, of Chicago, was re-elected secretary and treasurer.

PROFESSORS ERB, of Heidelberg, and LIEBERMEISTER, of Tübingen, are both named for the chair of the medical clinic of Leipzig, made vacant by the death of Professor Wagner.

College of Physicians and Surgeons.—The graduating exercises of the College of Physicians and Surgeons were held in the Grand Opera House.

The following is the list of graduates:

| | |
|---|---|
| Wm. Orr. Anderson | David P. Hueston |
| Franklin M. Bailey | David T. Jones |
| Hermon R. Bulson | William F. Malone |
| H. Leslie Burrell | Fitch C. E. Mattison |
| John A. L. Bradfield | Ernst J. Miller |
| George C. Brengle | Robert R. Michael |
| S. W. Burson | L. Frank Myers |
| Isaac W. Brown | George M. Nesbit |
| A. W. Burrows | Oscar F. Pile |
| Milton F. Coe Ph. B. | Henry F. W. Petersen |
| Neil Cameron | Charles Stirling |
| Frank C. Cullen | Walter B. Stewart |
| William Wilson Coker | Otto W. Stair |
| Levi B. Casey | Schuyler Shidler |
| John Howard Davies | James T. Stanton |
| Frank E. Duckworth | Bruno von Schallern |
| William S. Fowler | J. Joseph Selbach |
| Clifford P. Fall | James G. Sinclair |
| David W. Feltenstein | William B. Towle |
| John F. Glover | John James Wood |
| Henry A. Holliday | Daniel Baldwin Wylie |
| B. Y. Harris | Howard Eugene White |
| Thomas J. Haines | Luther R. Williamson |

Medicine in Syria.—The Syrian Protestant College at Beirut, in its catalogue for 1886–87, reports 103 graduates in its medical and pharmaceutical departments since 1871.

---

Training-schools for Attendants in Asylums for the Insane.—In an address upon this subject, read before the Fourteenth National Conference of Charities and Correction, held in Omaha during the past year, Dr. Richard Dewey, superintendent of the asylum at Kankakee, advocated the giving of special facilities for the instruction of attendants in this important duty. Such a school was organized in the institution of which he is superintendent, and the first class has just graduated—thirty-two in number.

Professor H. A. Johnson, of this city, addressed the class, and impressed upon its members the importance of their calling, and dwelt upon the fact that nursing now has become a profession, and honorable as well as important and responsible, and that intelligent effort must take the place of mere sympathy manifested in unavailing and often harmful ways.

The Chicago Medical College.—The commencement exercises of the Chicago Medical College were held in Central Music Hall. The following is the list of graduates:

| | |
|---|---|
| Louis Becker | James F. Keeney, B.S. |
| Edward A. Bemis | Henry H. Mather, B.S. |
| Everett J. Brown | Charles H. Mayo |
| Henry B. Carriel, B.S. | Otto G. Miller |
| Henry A. Fink | Charles W. More |
| William R. Fringer | James J. Morgan, Ph. B. |
| Henry D. Gardner | Edwin A. Morse |
| Frank F. Gray, Ph.B. | Edward C. Morton |
| Louis L. Gregory, A.M. | William H. Parker |
| Winfield S. Hall, B.S. | Frank C. Sarrazin |
| Joseph L. Hancock | Willis D. Storer |
| Fred J. Hodges, B.S. | Thomas B. Swartz, A.M. |
| Bayard Holmes, B.S. | Edwin P. Taylor |
| Ole T. Hougen | Henry A. Vennema |
| Frederick R. Hunt | Charles B. Wagner |
| Charles G. Ives | William L. Warriner |
| Chas. W. Johnson, A.B. | Henry C. Whiting |

Honorary—Robert U. Chapman

---

Oxygenated Water in Diabetes.—A. Le Bloud recommends, in *L'Union Médicale*, 53, 1887, in diabetes, the use of water saturated with oxygen. He thinks that the increased quantity of oxygen furnished to the system will burn up the sugar. Senator, however, has already shown that such a view is erroneous.

---

Unique Surgical Cases.—At the recent meeting of German Scientists and Physicians in Wiesbaden, Dr. Dick mentioned the following cases in his surgical experience: 1. Evulsion of a tendon *in toto*, together with the 2d phalanx of the thumb. 2. A complicated fracture of the elbow, in which the biceps-tendon, with the vessels and nerves above the joint, were cut through by a sharp fragment of bone. 3. A longitudinal fracture of the femur running into the knee-joint. 4. A fracture of the styloid process. 5. A fracture of the 2d cervical vertebra, with recovery. 6. A hairy tumor of the cornea.

---

A tablet erected by the Alumni Associciation of the Bellevue Hospital Medical College, in memory of the late Professor Austin Flint, M. D., LL. D., was unveiled at the Carnegie Laboratory, in New York, on Saturday evening, March 10.

Annales du Laboratoire de l'Hospice des Quinze-Vingts.—This is a new and important periodical for ophthalmologists and pathologists, edited by Drs. Fieuzal and Haensell, and published by Delahaye et Lecrosnier, of Paris, for the purpose of clinical and pathological study of the specimens (1,700 enucleated bulbs) in the Quinze-Vingt Hospital.

---

Radical Cure of Hernia.—Of 97 patients with 121 hernias, Schwalbe, of Magdeburg, has cured 66 per cent. by subcutaneous and subfascial injection of a mixture of alcohol and boiling water.

